# Approach to the patient with thyroid storm

**DOI:** 10.1210/clinem/dgag054

**Published:** 2026-02-08

**Authors:** Peter A Kopp, Ilaria Giordani, Ulla Feldt-Rasmussen, Aurélie Forget-Renaud

**Affiliations:** Division of Endocrinology, Diabetes and Metabolism, University Hospital of Lausanne and University of Lausanne, Lausanne CH-1011, Switzerland; Division of Endocrinology, Diabetes and Metabolism, University Hospital of Lausanne and University of Lausanne, Lausanne CH-1011, Switzerland; Department of Growth and Reproduction, Rigshospitalet, Copenhagen University Hospital, Copenhagen DK-2100, Denmark; Institute of Clinical Medicine, Faculty of Health and Clinical Sciences, Copenhagen University, Copenhagen DK-2100, Denmark; Division of Endocrinology, Diabetes and Metabolism, University Hospital of Lausanne and University of Lausanne, Lausanne CH-1011, Switzerland; Division of Endocrinology, Department of Medicine, Hôpital Charles-Le Moyne, Greenfield Park, QC J4V 2H1, Canada

**Keywords:** thyrotoxicosis, hyperthyroidism, thyroid storm

## Abstract

Thyroid storm, or thyrotoxic crisis, is a rare but life-threatening hypermetabolic state arising from severe thyrotoxicosis with a high risk of multiorgan failure.

It typically occurs in individuals with untreated or poorly controlled hyperthyroidism, most often Graves’ disease, though various other disorders may also precipitate it. Common triggers include infection, surgery, trauma, abrupt discontinuation of antithyroid medication, severe emotional or physical stress, and iodine exposure.

Clinical manifestations involve hyperthermia, tachyarrhythmia, cardiac and respiratory failure, and hepatic dysfunction. Neurological and gastrointestinal symptoms, such as agitation, confusion, delirium, vomiting, diarrhea, and profuse sweating, are frequent. Diagnosis is clinical rather than biochemical, and scoring systems are used to support the diagnosis.

Thyroid storm is more common in females, with an incidence of approximately 0.2 to 1.1 per 100 000 in the general population and 4.8 to 6.3 per 100 000 among hospitalized patients. Without rapid intervention, mortality remains high (5 to 25%), primarily due to cardiovascular collapse or multiorgan dysfunction.

Management requires immediate supportive therapy, including cardiovascular stabilization, respiratory support, and temperature control, along with identification and treatment of precipitating factors. Pharmacologic therapy involves antithyroid agents to block thyroid hormone synthesis, beta blockers to counteract adrenergic effects, corticosteroids to reduce peripheral conversion and support adrenal function, and, when appropriate, iodine administration after thionamides.

Early recognition and intensive management are crucial to prevent fatal outcomes, highlighting the need for vigilance in patients with severe thyrotoxicosis or decompensated hyperthyroidism.

## Case presentations

### Case 1

A 76-year-old man with a two-year history of prior amiodarone exposure presented with a fever of 39.2 °C, tachycardia of 160 beats per minute (bpm), delirium, profuse diarrhea, jaundice, and bilateral basilar crackles suggestive of heart failure and pneumonia. Urgent transthoracic echocardiography (TTE) confirmed acute *Heart Failure with Reduced Ejection Fraction (HFrEF)* decompensation with a left ventricular ejection fraction (LVEF) of 20%, global hypokinesis, left ventricular dilation, and moderate mitral regurgitation.

Laboratory findings revealed thyrotoxicosis with a TSH <0.005 mUI/L (0.4-4 mU/L), free T4 > 100 pmol/L (12-21), T3 24 pmol/L (3.5-6). Anti-TSH receptor antibodies were negative. A thyroid ultrasound showed an enlarged gland of about 32 mL with heterogeneous hypoechoic areas but no nodules or hypervascularity. Liver function tests revealed hepatic dysfunction with aspartate transaminase (AST) 250 U/L (8-33), alanine transaminase (ALT) of 180 U/L (7-45), and bilirubin 3.2 mg/dL (0.1-1.2).

Chest X-ray showed bilateral infiltrates consistent with acute pulmonary edema overlaid with patchy consolidations suggestive of multifocal pneumonia. Comorbidities included longstanding HFrEF (mixed etiology: hypertensive, ischemic, alcoholic, with paroxysmal atrial fibrillation), chronic kidney disease, a 50-pack-year smoking history, risky alcohol use, and prostate cancer status post-radiotherapy.

### Case 2

A 72-year-old male patient was admitted to a rural health center in the early evening with acute confusion, palpitations, fever (39.5 °C), dyspnea, and diarrhea. He was recently hospitalized for abdominal pain, and contrast-enhanced CT of the abdomen revealed uncomplicated diverticulitis. The patient's vital signs revealed an irregular heart rate of 160 bpm, blood pressure was 95/55 mmHg, and his respiratory rate was 28 breaths per minute. The oxygen saturation was 90%. Physical exam revealed a large multinodular goiter, warm, moist skin, atrial fibrillation with rapid ventricular rate, and edema of the lower extremities. A chest X-ray documented pulmonary congestion and left tracheal deviation due to the goiter. Laboratory analyses showed an elevated troponin I, leukocytosis, mild transaminitis, and a suppressed TSH of <0.01 mU/L. Peripheral hormones (free T4 and free T3) were ordered.

Therapy was initiated with intravenous propranolol for rate control and furosemide. Aspirin was prescribed as antipyretic therapy, but no other cooling measures were undertaken. Oral methimazole (MMI) was not administered due to difficulty swallowing, but Lugol solution at a dose of 10 drops was started. Transfer to a tertiary referral center was planned for the next morning. Within 12 hours, the patient developed progressive hypotension, acidosis, and multiorgan dysfunction. He sustained cardiac arrest and could not be resuscitated.

Peripheral hormones, received on the following day, showed a free T4 of 61.2 pmol/L (10-22), and a free T3 of 9.1 pmol/L (3.5-6.5), confirming a thyrotoxic profile with a markedly elevated free T4 and a less strikingly increased free T3, possibly in the context of critical illness.

### Case 3

A 30-year-old female migrant suffered a cardiopulmonary arrest on the street. Cardiopulmonary resuscitation (CPR) was initiated within a minute by a first responder. On arrival of the ambulance, she was in ventricular fibrillation, and she was defibrillated. Heart rate was normal after 5 minutes of resuscitation. Given persistent impaired consciousness (Glasgow Coma Scale score 3/15), she was intubated before transfer to a tertiary center. In the emergency room, she was hemodynamically stable, but she had atrial fibrillation with a tachycardic ventricular response. The ECG found no evidence of STEMI (ST-elevation myocardial infarction). A computer tomography (CT) of the brain ruled out intracranial bleeding. Thoracic CT showed no pulmonary embolism but revealed right ventricular enlargement, an enlarged pulmonary trunk, and ground-glass opacities suggestive of pulmonary edema. TTE showed mild right ventricular dilation and possible pulmonary hypertension. Troponine T was 11 ng/L (<14), creatinine kinase (CK) 86 U/L (25-140), and CK MB was 25 U/L (>6% of CK) with negative enzyme kinetics.

On admission, TSH was <0.005 mU/L (0.27-4.2), free T4 was 89.1 pmol/L (11.2-24.1), and free T3 was 32.6 pmol/L (3.1-6.8). Therapy was initiated immediately with PTU 200 mg every 6 hours, initially per nasogastric tube. About 1 hour later, she received a first dose of 65 mg of potassium iodide; it was then administered every 8 hours. Hydrocortisone 100 mg was given i.v. every 8 hours. Beta blockade was commenced with labetolol 5 mg i.v. and then at a rate of 0.5 mg/min. It was switched to propranolol 60 mg every 6 hours after day 1. After extubation, cholestyramine 4 g was given 3 times daily. Free T3 was in the reference range after 3 days (6.8 pmol/L), and both peripheral hormones were within the reference range after 10 days, with a free T4 of 24 pmol/L and a free T3 of 3.5 pmol/L.

Anticoagulation was started with heparin i.v. and later changed to oral apixaban.

A non-urgent coronary angiography was negative for coronary artery disease and cardiac magnetic resonance imaging (MRI) did not find alterations suggestive of cardiomyopathy.

The personal medical history obtained after the acute event revealed that she had been diagnosed with Graves’ disease at age 23 and treated with carbimazole (CMZ), but the patient had stopped the medication about 4 months earlier.

Follow-up TTE was performed one month later and found no evidence of pulmonary hypertension. Outpatient cardioversion for rhythm control was successfully performed 2 months after admission.

## Thyroid storm

**Figure 1:**
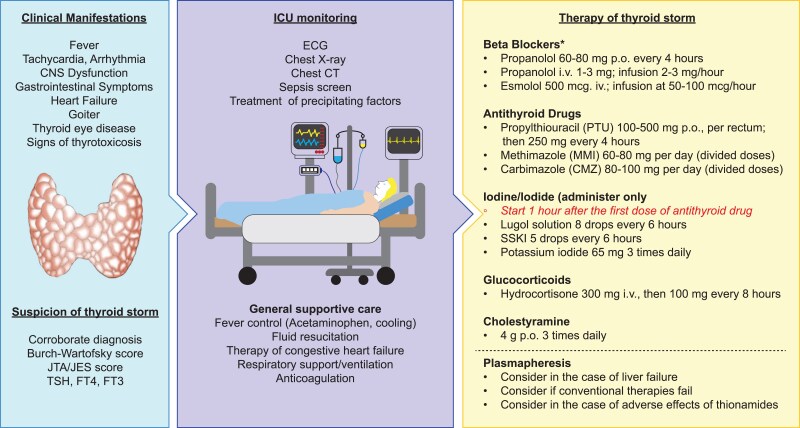
Diagnosis and management of thyroid storm. For beta-blockers, thyroid medications, and iodine, one of the listed options is chosen. *In addition to these commonly used beta blockers, alternative regimens with atenolol, metoprolol, and nadolol per os, as well as landiolol and labetalol intravenously, have been used successfully. For further details, see text and references (5, 6). Abbreviations: CT, computer tomogram; JTA/JES, Japan Thyroid Association/Japan Endocrine Society; p.o., per os; i.v., intravenous; SSKI, saturated solution of potassium iodide.

Thyroid storm is a critical, potentially life-threatening hypermetabolic condition caused by severe manifestations of thyrotoxicosis (1-4). It requires swift detection and emergency treatment to lower the risk of fatal outcomes (Fig. 1).

**Figure 2:**
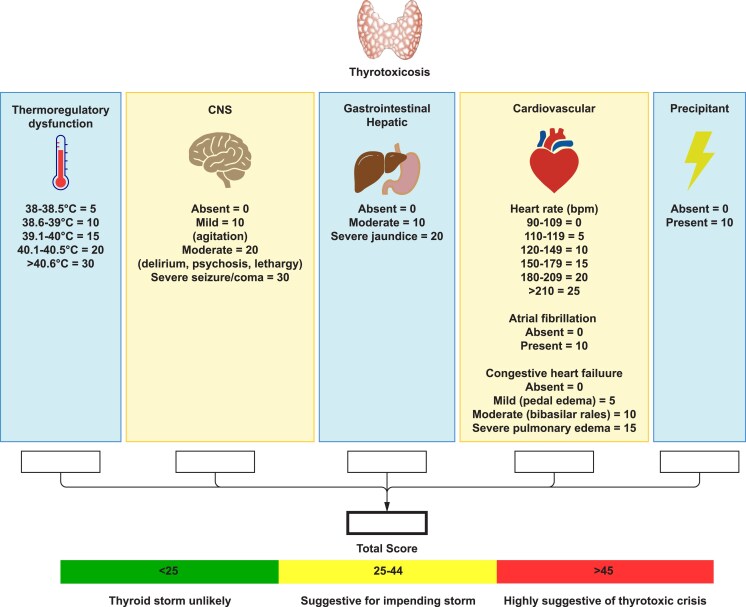
Graphical illustration of the Burch–Wartofsky scoring system for the diagnosis of thyroid storm. The Burch–Wartofsky Point Scale was first published in 1993 (1). CNS, central nervous system.

**Figure 3:**
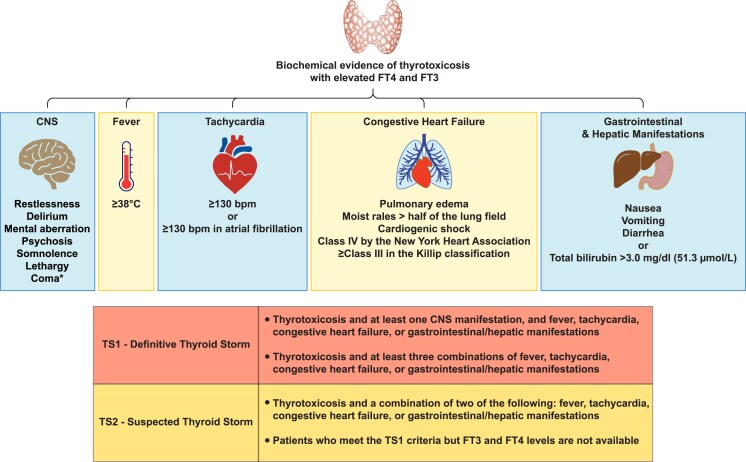
Graphical illustration of the Japan thyroid association/Japan endocrine society scoring system for the diagnosis of thyroid storm. The diagnostic criteria by the JAS/JES for thyroid storm were developed based on national surveys published in 2012 (7), and summarized in formal guidelines in 2016 (5). CNS, central nervous system; *: ≥1 on the Japan Coma Scale or ≤14 on the Glasgow Coma Scale; bpm, beats per minute; FT3, free triiodothyronine; FT4, free thyroxine).

The diagnosis of thyroid storm is based on the constellation and severity of clinical manifestations, as well as the functional decompensation of one or more organ systems, and is facilitated by 2 scoring systems (1, 7, 8) (Fig. 2 and 3). Based on standard thyroid function tests, most patients experiencing a thyrotoxic crisis cannot be differentiated from those with uncomplicated thyrotoxicosis.

Thyroid storm is frequently triggered by precipitating factors such as infections, including sepsis, surgical procedures, or discontinuation of anti-thyroid medication. However, in some patients, no clear precipitating event can be identified.

Graves’ disease is the most frequent underlying cause of thyroid storm. In some patients, thyroid storm is the initial presentation of Graves’ disease, and it is not uncommon that it is caused by discontinuation of anti-thyroid medications. Besides Graves’ disease, thyroid storm may also develop in patients with other etiologies of thyrotoxicosis, such as toxic multinodular goiter, toxic adenoma, or amiodarone-induced thyrotoxicosis (3, 7, 9-11). Very rarely, it has been reported in the context of subacute thyroiditis, hydatidiform mole, metastatic hormone-secreting thyroid carcinoma, therapy with tyrosine kinase or check-point inhibitors, or after ingestion of thyroid hormone tablets (3, 5, 12).

### Incidence and mortality

Estimates of the incidence rates of thyroid storm vary because of the heterogeneity in diagnostic criteria, and possibly due to geographic variation associated with nutritional iodine intake and factors determined by the health care system. Overall, the incidence ranges from 0.2 to 1.1 per 100 000 persons annually in the general population, and 4.6 to 6.3 per 100 000 persons per year for hospitalized patients according to studies published between 2016 and 2025 (Table 1) (9, 13-20). In accordance with the higher prevalence of autoimmune thyroid disorders in women, there is a 2:1 to 3:1 ratio of females to males. Although mortality rates have decreased with structured care, older patients with comorbidities and those receiving suboptimal management continue to face a significantly elevated risk of fatal outcomes that can be as high as 30% (14, 15, 20, 21). The primary causes of mortality in thyroid storm are multiorgan failure, congestive heart failure, tachyarrhythmias, respiratory failure, hypoxic brain syndrome, disseminated intravascular coagulation, and concomitant sepsis (7, 22). Thyroid storm appears to be more prevalent in summer (16, 17).

**Table 1:** Reported range estimates for incidence, sex ratio, and mortality

Incidence (per 100 000 persons/year)	0.2 to 1.1 (general population) 4.8 to 6.3 (hospitalized patients)
Male–female ratio	Approximately 1:2 to 1:3
Mortality	5% to 12% generally, Up to 30% in older adults and patients with comorbidities

Estimates based on studies published between 2016 and 2025 (9, 13-20).

In a retrospective Japanese national inpatient database analysis including 1324 patients with thyroid storm diagnosed between 2011 and 2014, the incidence was 0.2 per 100 000 per year, 71.3% were female, and the in-hospital mortality was 10.1% (17). Mortality was significantly associated with older age (≥60 years), neurological impairment at admission, lack of antithyroid and beta-blocker therapy, and need for mechanical ventilation and plasma exchange along with hemodialysis (17).

A retrospective study assessing the national incidence patterns of thyrotoxicosis with and without thyroid storm, as well as clinical outcomes among hospitalized patients in the United States from 2004 to 2013, identified 19 723 patients with thyroid storm (15). The annual incidence varied between 0.57 and 0.76 per 100 000 individuals in the general population, and between 4.8 and 5.6 per 100 000 in hospitalized patients. Thyroid storm was linked to a significantly increased hospital mortality, ranging from 1.2% to 3.6%, compared to 0.1 to 0.4% in patients with thyrotoxicosis without storm. Hospitalization costs adjusted for inflation rose significantly in patients with thyroid storm from $9942 to $12 660 from 2004 to 2013 (*P* < .01) (15). A retrospective multicenter study involving patients admitted to 31 intensive care units (ICUs) in France over an 18-year span (2000 to 2017) included 92 patients with thyroid storm (9). The mortality rate in the ICU was 17%, and the 6-month postadmission mortality rate was 22%. In multivariable analyses, in-ICU mortality was associated with sequential organ failure without cardiovascular component (odds ratio, 1.22; 95% CI, 1.03 to 1.46; *P* = .025) and cardiogenic shock occurring within 48 hours post-ICU admission (odds ratio, 9.43; 1.77 to 50.12; *P* = .008) (9). In a nationwide retrospective population-based study utilizing the Taiwan Health and Welfare Data, 1244 patients with thyroid storm and 83 874 patients with thyrotoxicosis without storm were included, resulting in a thyroid storm prevalence of 1.48% (1244/83 874) (16). The incidence was 0.55 per 100 000 individuals per year, and 6.28 per 100 000 yearly in hospitalized patients. Most thyroid storm patients were female (67.9%) with ages ranging from 30 to 44 years (33.4%). Similar to a previous Japanese study (17), most thyroid storm admissions occurred in summer. Advanced age, male sex, pre-existing health conditions, ischemic stroke, myocardial infarction, and heart failure were associated with a significantly higher mortality risk (16). Data from the German claims database identified 1690 thyroid storm cases from 2007 to 2017 (19). Among all patients experiencing thyroid storm, 72% were women, and their average age was 60 years. The annual incidence of thyroid storm was 1.4 cases per 100 000 women and 0.7 per 100 000 men. The thyroid storm rate was higher in those over 60, with 2.7 cases per 100 000 women and 1.7 cases per 100 000 men annually. Thyroid storm mortality was low in patients under 60 years, at 1.4% for women and 1.0% for men; the likelihood of fatal outcomes rose significantly in individuals over 60 years, with rates of 10.9% for women and 16.7% for men (19).

In a prospective multicenter study conducted in Japan to assess the impact of the 2016 Guidelines for thyroid storm management by the Japan Thyroid Association and Japan Endocrine Society (JTA/JES) (5), 110 patients were included (14). The 30-day mortality rate was 5.5%, about half of the 10.7% reported in a prior nationwide survey (7), suggesting a beneficial impact of the guidelines. Patients with an APACHE II score ≥12 (23), who were not treated according to the guidelines, had a significantly higher mortality rate (50%) compared to those treated per guidelines (4.7%) (*P* = .01) (14).

A retrospective analysis of the United States National Inpatient Sample Population covering 2016 to 2020 found that 650 974 patients were admitted for thyrotoxicosis, with 16 175 experiencing thyroid storm (2.5%) (18). The incidence rose from 0.91 per 100 000 in 2016 to 1.03 per 100 000 in 2020. In contrast to the Japanese data (14), the mortality rate increased from 2.9% (2016) to 5.3% (2020) (*P* < .001) (18). An independent retrospective study using the National Inpatient Database (2016 to 2020) examined hospitalization trends, outcomes, and healthcare burden for thyrotoxicosis, with and without thyroid storm (20). A total of 33 430 hospitalizations were analyzed. Hospitalizations for thyrotoxicosis declined from 7444 in 2016 to 5424 in 2020. However, individuals who were admitted faced an elevated risk of mortality, regardless of the presence or absence of thyroid storm (without storm (10 [0.17%] in 2016 and 55 [1.30%] in 2020; *P*_trend_ < .001); with thyroid storm (10 [0.62%] in 2016 and 50 [4.15%] in 2020; *P*_trend_ = .051)). The length of hospital stays rose, and inflation-adjusted costs increased (2016: $36 408; 2020: $49 031; *P*_trend_ < .001). Finally, thyroid storm was associated with higher odds of major adverse cardiovascular events (MACE) (20).

Treatment and management of patients with thyrotoxicosis appear to be impacted by socioeconomic factors (24). Compared to individuals with commercial insurance, patients without health insurance were found to have a 12-fold higher risk of hospitalization for thyrotoxicosis and, among them, the prevalence of thyroid storm was very high at 32% (24). Additionally, higher income and education were associated with a decreased rate of hospitalization for thyrotoxicosis.

### Historical aspects

Jean-François Coindet, who practiced in Geneva, Switzerland, successfully employed potassium iodide for goiter treatment around 1819. Its effectiveness in this iodine-deficient region led to its widespread use, but by 1821, he reported that several patients experienced adverse events, especially severe tachycardia, one of the first descriptions of the cardinal symptoms of hyperthyroidism (25). Others confirmed this observation and Jean-Pierre Colladon reported a fatal event, perhaps the first report of death due to a thyrotoxic crisis (26). This form of thyrotoxicosis, arising in multinodular goiters with autonomous nodules, was later described as “*Jod-Basedow”* by Theodor Kocher in 1910 (27).

In the 1920s, Frank Howard Lahey, a prominent surgeon in Boston, drew attention to the risk of the “*crisis of exophthalmic goiter”* and the associated post-surgical mortality (28). He concluded that “*Thyroid crises are by no means rare”* and that “*The mortality rate in patients permitted to advance into a state of crisis is extremely high”*. At that time, thyroid surgery was a common precipitant of thyroid storm in patients with overt hyperthyroidism. Around the same time and based on the erroneous hypothesis that Graves’ disease represents a form of “*dysthyroidism’’* in which the gland releases an atypical thyroid hormone or toxin, Henry Plummer prescribed Lugol's solution (a mixture of potassium iodide and iodine) to patients with exophthalmic goiter scheduled for thyroidectomy. Remarkably, and in contrast to the aggravation seen in patients with toxic multinodular goiters, this treatment improved the manifestations of hyperthyroidism, often resolving them within days (29). Moreover, the surgical mortality rate decreased from approximately 4% to 5% at the Mayo Clinic to less than 1%. Besides the effect of iodine on thyroid blood flow (30, 31), a reduction or normalization of peripheral thyroid hormone levels likely contributed to the improvements in outcomes. This so-called Plummer effect is explained by the fact that high intrathyroidal iodide concentrations inhibit hormone release in individuals with Graves’ disease, a key mechanism for treating thyroid storm (see below) (32, 33). Interestingly, the observation that iodine therapy improved symptoms in patients with “*exophthalmic goiter”* had been made even earlier by Trousseau in 1862 (34).

The introduction of thiourea and thiouracil drugs for the control of hyperthyroidism by Ted Astwood in the early 1940s fundamentally transformed the treatment of thyrotoxicosis (35-37), including thyroid storm (38). The development of beta blockers such as propranolol in the mid-1960s by Sir James Black (Nobel prize 1988) also had a fundamental impact on the management of thyrotoxicosis and reducing the associated mortality (39).

## Clinical presentation and diagnosis

The medical history can suggest thyroid storm by identifying classic symptoms associated with thyrotoxicosis, a history of hyperthyroidism, discontinuation of antithyroid medications, iodine exposure, infections, recent surgery, acute illness, or major emotional stress (1-3).

Most patients with thyrotoxic crisis show characteristic signs of thyrotoxicosis, including goiter with or without nodules, and, in patients with Graves’ disease, signs of thyroid eye disease. The spectrum of manifestations varies in intensity and combination (3, 40, 41). Key clinical features include high fever (often >38.5 °C), tachycardia disproportionate to the degree of fever, arrhythmias such as atrial fibrillation, congestive heart failure, or pulmonary edema, changes in mental status from agitation to confusion or coma (21), and gastrointestinal disturbances such as nausea, vomiting, diarrhea, or jaundice (42). Although more common in older adults, reversible dilated cardiomyopathy can also occur in younger thyrotoxic patients, and Takotsubo cardiomyopathy has also been associated with thyrotoxic crises (3).

Rarely, and more commonly in older patients, classical signs and symptoms of thyrotoxicosis are absent, a situation designated as “*apathetic hyperthyroidism.”*

The diagnosis of thyroid storm is primarily clinical and supported by scoring systems, the Burch–Wartofsky Point Scale, first published in 1993 (1, 8) (Fig. 2), and the criteria developed by the JTA/JES (5, 7) (Fig. 3). The Japanese criteria were proposed in 2012 in the context of a nationwide survey on the incidence of thyroid storm (7), and they were subsequently published alongside guidelines for managing the condition (5).

The Burch–Wartofsky scale assigns points based on clinical signs, including temperature, central nervous system involvement, heart rate, gastrointestinal symptoms, heart failure, arrhythmias, and precipitating factors. Similarly, the JTA/JES criteria combine clinical and laboratory findings to support the diagnosis. A Burch and Wartofsky score <25 points suggests that thyroid storm is unlikely, a score of 25 to 44 points is suggestive of a possible impending crisis, and >45 is highly suggestive of a thyroid crisis. The JTA/JES scoring system categorizes patients as having either Definite Thyroid Storm (TS1) or Suspected Thyroid Storm (TS2) (5). The 2 systems have been shown to accurately identify most patients with thyroid storm (7, 40, 42). Furthermore, the scores are predictive of mortality as well as length of hospital and ICU stay (40). Even though these tools aid in diagnosing thyroid storm, clinical judgment remains essential. Thyroid storm during pregnancy has been reported but is exceedingly rare (43).

### Laboratory findings

Serum total and free T4 and T3 concentrations are elevated but not necessarily higher than in uncomplicated thyrotoxicosis (44, 45). Serum T3 can occasionally be misleading and be within the reference range because of reduced peripheral conversion in the context of severe illness (3). Higher APACHE II and SOFA scores (23, 46), reflecting more severe illness, were strongly linked to increased patient mortality, while FT4, FT3, and the FT3/FT4 ratio were not (47). Lower FT3 and FT3/FT4 ratios are associated with greater disease severity, suggesting inhibited T4-to-T3 conversion in severely ill patients (47).

Hyperglycemia, elevated lactate, leukocytosis without infection, and mild hypercalcemia are common. Transaminases and alkaline phosphatase may be elevated. Serum cortisol should be high during acute illness; low levels are suggestive of adrenal insufficiency or reduced adrenal reserve (48).

### Differential diagnosis

Key differential diagnoses of thyroid storm include controlled thyrotoxicosis, sepsis, encephalitis or meningitis, hypertensive encephalopathy, heatstroke, drug intoxication (eg, cocaine or amphetamines), and neuroleptic malignant syndrome (49). These conditions can present with symptoms similar to thyroid storm and should be considered and ruled out during evaluation.

## Pathogenesis

The severity of thyroid hormone elevation alone does not explain thyrotoxic crisis (3). Some patients with very high hormone levels remain compensated, whereas others with more modest hormone levels develop a crisis (11). Concomitant precipitating factors are frequently but not always identifiable. Although symptoms resemble catecholamine excess, actual catecholamine levels are typically normal, suggesting that increased tissue responsiveness and β-adrenergic receptor activity play key roles, explaining the clinical efficacy of beta-blockers. The mechanisms underlying why only a subset of thyrotoxic patients progress to thyroid storm despite comparable hormone levels remain incompletely understood (13). Precipitating factors likely provoke rapid hormone surges or heightened tissue sensitivity through mechanisms such as increased cytokine release, variations in adrenergic receptor responsiveness, and comorbidities such as heart failure or infection, which may amplify the hypermetabolic response (11, 13, 50). Additionally, cellular metabolic stress and uncoupling of oxidative phosphorylation during conditions like hypoxia or infection may increase thermogenesis and systemic symptoms (3).

## Treatment of thyroid storm

Given the mortality risk of untreated thyroid storm, clinical features such as goiter, high fever, marked tachycardia, and a precipitating illness warrant prompt treatment initiation even before test results become available. The treatment is multimodal and aims at reversing the severe systemic effects of thyroid hormone excess, as well as the manifestations of (multi)organ involvement and failure. Key principles of therapy are summarized in the *2016 American Thyroid Association Guidelines for Diagnosis and Management of Hyperthyroidism and Other Causes of Thyrotoxicosis* (6), and very detailed recommendations are provided in the *2016 Guidelines for the management of thyroid storm from JTA/JES* (2, 5, 51). The *2018 European Thyroid Association Guideline for the Management of Graves’ Hyperthyroidism* provides a succinct overview but does not comment on the use of iodine as a therapeutic intervention (51, 52).

### Supportive therapy

Patients should be cared for in an ICU for continuous monitoring and treatment. Initial treatment must address cardiovascular complications, heart rate control, and respiratory failure (3, 5, 6, 52). Hyperthermia must be treated with cooling measures and antipyretic therapy (paracetamol). Aspirin is contraindicated because it displaces thyroid hormones from binding proteins, increasing free hormone levels, thereby worsening thyrotoxicosis (53). Chlorpromazine may be used if needed to reduce agitation and fever (10). Corticosteroids are administered to reduce peripheral thyroid hormone conversion and mitigate potential overt or partial adrenal insufficiency (54, 55). Electrolyte disturbances in thyroid storm are manifold, including hyponatremia from hypovolemia due to diaphoresis, vomiting, or diarrhea; hypomagnesemia from gastrointestinal losses; and hypo- or hyperkalemia. Hypokalemia arises from increased sympathetic activity driving intracellular potassium shifts via beta-2 adrenergic stimulation of Na^+^/K ^+^ -ATPase, while hyperkalemia may be associated with acidosis. Acid–base disturbances such as metabolic acidosis or respiratory alkalosis, and the associated electrolyte imbalances, must be promptly identified and corrected (56). Blood glucose levels should be closely monitored due to hypoglycemia risk. Nutritional support including vitamin replacement (like thiamine) may be necessary.

### Treatment of underlying precipitating conditions

Timely identification and management of infections and sepsis as classic precipitating factors are crucial to control thyrotoxic crisis.

### Beta blockade

Beta blockers are essential in thyroid storm management by blocking the increased sympathetic activity and controlling manifestations such as tachycardia, anxiety, and tremors. Propranolol is recommended at a dose of 60 to 80 mg every 4 hours for thyroid storm, as per ATA and ETA guidelines (6, 52). This not only controls symptoms such as tachycardia but may also reduce the conversion of T4 to T3. For patients who are unstable or unable to take oral medication, intravenous administration is an option (Propanolol: slow bolus 1 to 3 mg at a rate not exceeding 1 mg/min i.v.; then infusion at 2 to 3 mg/hour; Labetalol: 5 to 20 mg i.v. over 2 minutes; infusion 0.5 mg/min). The JTA/JES guidelines recommend using beta1-selective beta blockers such as landiolol or esmolol intravenously, and bisoprolol orally, favoring them over propranolol due to a retrospective analysis suggesting increased mortality in heart failure patients treated with propranolol (5, 47). However, a subsequent retrospective cohort study found similar in-hospital mortality rates between patients treated with propranolol and those receiving beta1-selective beta blockers (7.4% vs 6.3%), even in cases of acute heart failure (57).

In patients with suspected or known congestive heart failure, invasive monitoring and cautious use of lower initial beta-blocker doses are advised because beta-blockade can cause cardiovascular collapse (58). Esmolol (500 mcg/kg i.v. over 1 minute; infusion at 50 to 100 mcg/kg/minute), an intravenous beta-blocker with a very short half-life of approximately 9 minutes, is often preferred, as it allows rapid discontinuation if adverse effects occur (5, 6, 59). If beta-blockers are contraindicated, such as in asthma or cardiac decompensation, diltiazem may be used as an alternative (60).

### Reduction of thyroid hormone production and secretion

Antithyroid medications such as propylthiouracil (PTU), MMI, or its precursor CMZ are used to inhibit thyroid hormone synthesis (36, 37). PTU is often preferred in thyroid storm because it additionally inhibits the peripheral conversion of T4 to T3. However, in a recent comparative effectiveness study of a multicenter cohort including 1383 adult patients with thyroid storm (656 treated with PTU, 727 with MMI), no significant differences were found in mortality or adverse events among patients treated with the 2 drugs (61). In a Japanese survey including 356 thyroid storm patients, MMI was used preferentially (276 patients) and showed no disadvantages compared to PTU (45 patients) (47). PTU is typically started with a loading dose of 500 to 1000 mg given orally, via nasogastric tube, or per rectum (62-64), followed by 250 mg every 4 hours. MMI can be prescribed at 60 to 80 mg/day or CMZ at 80 to 100 mg/day. In cases of disturbed liver function (when bilirubin and liver enzymes are 3 times the upper limit of normal), MMI or CMZ are favored over PTU. Intravenous MMI is available in parts of Europe and Japan (47), but not in the United States (65), and intravenous PTU is no longer commercially available. A method for preparing an MMI solution has been published (65). The doses should then be tapered according to peripheral hormone concentrations.

Iodine inhibits synthesis (Wolff–Chaikoff effect) and release of thyroid hormones (Plummer effect) (32, 66-68). It should be administered at least 1 hour after giving antithyroid drugs, although it has been used successfully in drug-naïve patients with thyrotoxicosis caused by Graves’ disease (29, 69-71). The delay in administration is, however, crucial to prevent worsening of thyrotoxicosis through an increase in substrate for hormone synthesis in patients with nodular goiters harboring autonomous nodules. Typical regimens include Lugol's iodine (8 drops 4 times per day (A 5% Lugol's solution delivers approximately 6.25 milligrams of total iodine per drop (0.05 mL), composed of about 2.5 mg elemental iodine (I2) and 3.75 mg potassium iodide.)) or a saturated solution of potassium iodide (SSKI, 5 drops 4 times per day (35 to 50 mg iodide/drop.)), or potassium iodide tablets (65 mg 3 times daily). They should be discontinued once the patient stabilizes. To minimize local irritation, iodine solutions should be diluted in fluids such as milk or juice before oral or nasogastric administration (72, 73).

Steroids play a crucial role in the treatment of thyroid storm by decreasing the peripheral conversion of T4 to T3. Additionally, steroids help prevent or treat overt or relative adrenal insufficiency, which can occur due to the hypermetabolic state induced by excess thyroid hormones. The typical regimen includes intravenous hydrocortisone with a loading dose of 300 mg, followed by 100 mg every 8 hours. Steroid therapy is particularly beneficial when Graves’ disease is the underlying cause of thyroid storm. Treatment should continue until clinical improvement occurs, with tapering thereafter.

Perchlorate (a competitive inhibitor for iodide uptake by the sodium-iodide symporter) and iopanoic acid (an inhibitor of the 5′-deiodinase enzymes deiodinase type 1 and 2 converting T4 to T3) have been used in the therapy of thyroid storm, but they are no longer readily available. Further adjuncts include cholestyramine 4 g 3 times daily, which increases thyroid hormone clearance by blocking enterohepatic circulation (an off-label use), and lithium, an inhibitor of thyroid hormone secretion, which can be considered with specialist advice due to toxicity risks (11, 74).

### Adjunct therapies

Plasmapheresis can be utilized in rare circumstances when conventional therapies fail, if thionamide therapy is contraindicated because of liver failure, severe adverse reactions to thionamides (75), or when rapid preoperative stabilization is needed (76). Its rationale lies in the ability to quickly remove circulating thyroid hormones and cytokines from plasma, resulting in temporary but significant reductions in hormone levels (47, 77). The JTA/JES guidelines offer detailed recommendations when plasmapheresis can be considered (5).

Anticoagulation is recommended for patients with persistent atrial fibrillation based on their CHADS2 score (78), a validated tool used to stratify stroke risk (5, 6, 52).

### Surgery

Surgical thyroidectomy for thyroid storm is generally not recommended before thyrotoxicosis is controlled medically (3, 49). However, some have advocated for surgery if standard medical treatment fails to improve the patient within 12 to 24 hours (79). This approach remains controversial and should be reserved for very rare situations, such as severe allergy to antithyroid drugs or urgent need for rapid resolution. Overall, thyroidectomy is a secondary option when aggressive medical therapy is insufficient.

### Long-term therapy

Once stabilized, patients are maintained on antithyroid medications and are generally advised to undergo definitive treatment for hyperthyroidism, such as radioactive iodine therapy or thyroidectomy, to prevent recurrence (5).

## Back to the cases

### Case 1

This case illustrates thyroid storm, scoring 65 points on the Burch–Wartofsky scale and meeting Japanese Thyroid Association (JTA) TS1 criteria (1, 5, 8), with multiorgan failure (cardiovascular, central nervous system, gastrointestinal/hepatic, pulmonary), potentially triggered by bilateral pneumonia. The etiology is compatible with amiodarone-induced thyrotoxicosis (AIT) secondary to destructive thyroiditis (AIT type 2), and possible focal zones of autonomy (AIT type 1) (80).

Initial ICU management included loop diuretics alongside inotropic support with dobutamine, cooling, and i.v. antibiotics. Initial thyrotoxicosis treatment comprised i.v. propranolol (1 to 3 mg every 4 to 6 hours), PTU (200 mg every 8 hours), hydrocortisone (100 mg every 8 hours), and cholestyramine. After 4 days, therapy was switched to CMZ 20 mg and prednisone 20 mg per os. Full normalization of peripheral hormones was only achieved after 4 months of therapy. Definitive treatment then consisted of total thyroidectomy under general anesthesia; total thyroidectomy under loco-regional cervical anesthesia with sedation or hypnosis, an option in selected high-risk patients (81), has been considered as an alternative (82).

### Case 2

This vignette illustrates several critical management failures (5, 6, 52). Although a thyrotoxic crisis was considered likely, and later confirmed by the biochemical constellation, treatment did not adhere to published recommendations, expert consultation was not promptly obtained, and transfer to a tertiary care center was not initiated immediately. Elevated troponin, pulmonary congestion, hypotension, and hypoxia indicate multiorgan involvement and possible cardiogenic shock requiring urgent intervention. Close hemodynamic and respiratory monitoring was not possible in this rural hospital. Aspirin was used for fever control, which may have increased thyroid hormone levels. Oral CMZ was not administered due to swallowing difficulty but no alternative routes were utilized. Lugol solution was started without prior thionamide therapy, providing further substrate for thyroid hormone synthesis in this patient with a multinodular goiter in whom the administration of contrast medium during the evaluation for diverticulitis may have been a triggering factor. Finally, glucocorticoids were not administered to inhibit peripheral conversion of thyroid hormones and to provide protection against potential adrenal insufficiency. The fatal outcome likely resulted from the combination of suboptimal treatment, inadequate monitoring, and incorrect medication choices in this critically ill patient with thyroid storm.

### Case 3

This patient with Graves’ disease, who had been non-adherent to therapy, exemplifies excellent management of thyroid storm complicated by cardiac arrest. It also illustrates that cardiovascular complications can occur in young patients (83). The therapeutic approach was fully aligned with current recommendations and guidelines (5, 6, 52). At initial presentation, immediate bystander CPR and rapid defibrillation for ventricular fibrillation led to the timely restoration of circulation. This was followed by early intubation because of impaired consciousness, followed by transfer to a university center for advanced monitoring and care. In the tertiary center, thyrotoxic crisis was immediately recognized and combined with a comprehensive diagnostic workup excluding other causes for cardiac arrest (STEMI, pulmonary embolism, intracranial bleeding). ICU monitoring allowed for close hemodynamic surveillance and respiratory support, and care was coordinated through a multidisciplinary approach. Anticoagulation with heparin and apixaban was appropriate for atrial fibrillation post-cardiac arrest and right ventricular dysfunction.

Therapy for thyroid storm was initiated promptly with PTU per nasogastric tube, followed by the administration of potassium iodide. Beta-blockade was initiated with labetalol i.v., then transitioned to propranolol, effectively controlling adrenergic symptoms and tachyarrhythmia. In addition to administering hydrocortisone i.v., the patient was also given cholestyramine to interrupt enterohepatic recycling of thyroid hormones. This case also illustrates that these measures lead to a rapid normalization of the peripheral hormones. Coronary angiography was deferred until patient stabilization through intensive therapy of thyrotoxicosis, given the contraindication of iodinated contrast during untreated thyroid storm (84).

Nonadherence to CMZ was likely the major precipitating factor for the thyrotoxic crisis. This migrant patient, with limited resources, poor socioeconomic integration, and linguistic barriers, also exemplifies that vulnerable populations are at increased risk for developing thyroid crises.

## Conclusions

Guideline-directed therapy for thyroid storm generally enables effective control of thyroid storm by targeting multiple pathogenic pathways through the combined use of antithyroid drugs, iodine, beta-adrenergic blockers, corticosteroids, and cholestyramine. Early recognition of the possibility of a thyrotoxic crisis is crucial and can be supported by validated scoring systems. Prompt initiation of a multimodal treatment in an intensive care setting, alongside management of precipitating factors, attenuates the risk of multisystem organ failure and has markedly reduced the fly high mortality rate associated with thyroid storm.

## Data Availability

Data sharing is not applicable to this article as no datasets were generated or analyzed for this study.
